# Exploring alternative financing models and early access schemes for orphan drugs: a Belgian case study

**DOI:** 10.1186/s13023-022-02571-8

**Published:** 2022-12-09

**Authors:** Khadidja Abdallah, Kathleen Claes, Isabelle Huys, Lennert Follon, Charlotte Calis, Steven Simoens

**Affiliations:** 1 Department of Pharmaceutical and Pharmacological Sciences, KU Leuven, Onderwijs en Navorsing 2 Bus 521, Herestraat 49, 3000 Louvain, Belgium; 2 Nephrology and Renal Transplantation Research Group, Department of Microbiology, Immunology and Transplantation, KU Leuven, Louvain, Belgium; 3 Department of Nephrology and Renal Transplantation, UZ Leuven, Louvain, Belgium

**Keywords:** Financing models, Insulated fund, Private insurance, Early access schemes, Compassionate use, Medical need, Off-label use

## Abstract

**Background:**

Although some jurisdictions have implemented particular adjustments to accommodate often-expensive orphan drugs in their healthcare systems, availability of these drugs remains complex. This study investigates alternative financing models and early access schemes for orphan drugs in the context of the Belgian healthcare system.

**Methods:**

Three focus group discussions were held with a panel of eleven experts from the Belgian Drug Reimbursement Committee, the Colleges for Orphan Drugs, the pharmaceutical industry, physicians, ethicists and pharmacists. Retrieved data were pseudonymised, analysed and coded according to the Qualitative Analysis Guide of Leuven.

**Results:**

Experts disfavoured the insulated fund as well as private insurance for financing orphan drugs, as, respectively, isolation of a separate budget and a mostly profit-driven mechanism would contradict the Belgian fundamental principle of solidarity. Moreover, an insulated fund could, albeit on a smaller scale, reproduce the same budgetary constraints as the general reimbursement system. As the Special Solidarity Fund is intended for urgent care and exclusively accommodates financial needs subject to eligibility criteria, its design would not allow general financing of orphan drugs. Overall, implementation of an alternative financing model was not endorsed, instead, improving the current reimbursement system was preferred. Suggestions mentioned were; increased collaboration and transparency, robust and quality real-world evidence but also digitalization of data. Alleviating administrative burden and simplifying the admission process of compassionate use program, medical need program and early treatment reimbursement should be prioritized to facilitate early access. Furthermore, a legal framework for off-label use could stimulate proper implementation. Efforts on collaboration of expertise centres and coordination of orphan drug databases across Europe could foster a robust data network to support orphan drug availability in individual countries.

**Conclusions:**

This research reveals that reassessing current financing models and early access schemes by eliminating inadequacies, may be more conducive than establishing alternative systems to increase availability of orphan drugs in Belgium. Other jurisdictions may rely on this information to review their own models of early access and financing to cultivate a more sustainable delivery of orphan drugs.

**Supplementary Information:**

The online version contains supplementary material available at 10.1186/s13023-022-02571-8.

## Introduction

Pricing and reimbursement have been identified as key determinants for national access to orphan drugs [1–3]. This has led to discrepant accessibility of orphan drugs among EU Member States [4]. In 2020, 96% of EMA approved orphan drugs were available to patients in Germany whereas, in Latvia, this was as little as 2% [5]. Market authorization holders often focus on commercially viable and lucrative markets to obtain higher prices for their innovative orphan drugs [4]. As a result, poorer countries, unable to afford the suggested price, experience delays in access or do not offer these orphan drugs [4, 6].

To access high-cost orphan drugs people living with a rare disease rely on financial support [7–9]. It is, therefore, essential to minimize co-payments or fully reimburse orphan drugs by national healthcare systems or insurance funds. Many countries adopt health technology assessment (HTA) in which budget impact and cost-effectiveness are reoccurring components to evaluate a novel drug [6, 10]. Orphan drugs are often deemed not cost-effective and pressure-exerting on already overtaxed healthcare budgets [11]. Several studies also reported on the fast-growing share of orphan drugs in healthcare budgets compared to other drugs [12–14]. In this light, various countries have established methods to control budget spending and the opportunity cost associated with funding orphan drugs.

In Italy, the Agenzia Italiana del Farmaco (AIFA) fondo 5% functions as a financial source for not only independent research and development but also for funding requests of orphan drugs with pending authorization [15–17]. The fund raises its capital from pharmaceutical companies that contribute 5% of their promotional expenses. In Scotland, the New Medicines Fund is similar as it is financed by a proportion of pharmaceutical companies’ earnings [18]. The fund is intended to accommodate high cost orphan drugs denied or recommended for reimbursement by the Scottish Medicines Consortium [19, 20]. Built on the success of the Cancer Drug Fund, the United Kingdom recently established the Innovative Medicines Fund. The fund is a fixed budget for temporary and conditional access to advanced therapy medicinal products, including those for non-cancer rare diseases [21, 22]. Private health insurance is a well-known mechanism of financing drugs in the United States of America and Canada [23, 24]. Although private health insurance may reach a wider audience, it is often subject to malfunction due to poor information dissemination, income and knowledge inequity and misuse of market power [25].

To address financing limitations and accelerate access to orphan drugs with pending authorization or under development, several countries adopt alternative access schemes [26]. Examples are, compassionate use programs (CUP) provided by pharmaceutical companies and medical need programs (MNP), such as the French ‘Autorisation Temporaire d’Utilisation d’extension’ (ATU) [27–29]. In some instances, people living with a rare disease are treated with a medicinal product not yet indicated or authorized for their specific condition under, so-called, off-label use [30]. Even if the particular drug is considered safe, in life-threatening circumstances off-label use is often seen as a last resort due to its inherent experimental character.

In this study, we aimed to explore health economic, ethical, legal, social and practical aspects of aforementioned access schemes and financing models for orphan drugs. Although these issues were investigated in the setting of the Belgian health care system, the analysis provides additional insights in trade-offs and (dis)advantages that are likely to be relevant to other countries.

## Belgian financing models and access schemes for (orphan) drugs: the status quo

In the Belgian reimbursement process, orphan drugs benefit from a privileged position. These drugs are exempt from the claw-back measure [31], do not require pharmaco-economic evaluations and are often made available by means of a managed entry agreement despite clinical uncertainties [14]. Furthermore, approximately 30% of orphan drugs have an assigned Orphan Drug College which consists of expert-physicians who evaluate case-by-case eligibility and adoption rights [32, 33]. Among other responsibilities, the College also advises the consultant-physician of sickness funds on reimbursement of an orphan drug [34].

For non-traditional financing of urgent medical care, the Special Solidarity Fund (SSF) can function as a last resort for individuals that meet its strict criteria [35]. Eligibility is determined by the College of Medical Doctors Directors depending on urgency, safety, alternative treatments, critical medical need, scientific value and effectiveness [35]. However, the SSF only temporarily and, for adults, partially covers expenses as not only treatments beyond orphan drugs are considered but also medical devices and cross-border healthcare [35, 36]. In 2010, the Belgian Health Care Knowledge Centre drafted recommendations to resolve administration and complex operations of the SSF [36]. Improvements in terms of automatization and digitalization, oral communication with the Orphan Drug College and also transparent follow-up of dossiers were specified [36]. An additional way of non-traditional drug financing in Belgium is private health insurance. Unlike North-American systems, private coverage in Belgium strictly exists complementary to the mandatory public health insurance and amounts to just 5.1% of current healthcare expenditure [37, 38].

Early temporary authorization (ETA) in Belgium forms the cornerstone of early but regulated access to orphan drugs [31]. Under ETA, an innovative drug, with pending central authorization or not available through the conventional reimbursement system, can be granted to patients in critical medical need and with a seriously debilitating or life-threatening disease included on the National Institute for Health and Disability Insurance’s (NIHDI) ‘Unmet Medical Need’ list [39, 40]. Furthermore, an application for market authorization or clinical trials with preliminary evidence of the drug should be ongoing. A license for ETA is requested by the pharmaceutical company and depending on prior authorization in another indication or not, respectively, a medical need program (MNP) or a compassionate use program (CUP) could be initiated [39]. If the pharmaceutical company additionally applies for monetary compensation of CUP or MNP, it is defined as early temporary reimbursement (ETR). Another way of early access to orphan drugs is through off-label use. Although reports of side-effects exist and ethical approval of off-label use is required, only a handful of official guidelines have been formulated [30]. Moreover, unless sponsored through charity, reimbursement of off-label treatment is not provided by the Belgian state.

## Methods

### Study design

Focus group discussions were chosen as a qualitative explorative research technique to enable in-depth information gathering and interactions between participants. In light of national healthcare measures, a total of three discussions, of two and a half hours each, were held through Zoom videoconferences between October 2020 and February 2021. The moderation was led by pharmacy students, LF and CC, and supervised by SS and KA. In the first session, conversations were initiated by presenting stakeholders with predetermined questions based on a literature review (see Additional file 1). In subsequent focus group discussions, key topics that were highlighted prior were elaborated further and posed questions were adjusted to stakeholders’ previous remarks (see Additional file 1). The study was approved by the KU Leuven Ethics Committee on September 9th, 2020 (reference n^o^ MP015702).

### Participants

Each focus group discussion consisted of eight to eleven representatives of the Belgian Drug Reimbursement Committee, the Colleges for Orphan Drugs, the pharmaceutical industry, physicians, ethicists and pharmacists. Candidates were selected based on Belgian nationality and expertise in payment and access of orphan drugs. Invitations were sent by e-mail followed by a reminder a week after. Respondents were provided with arrangements on the time and course of scheduled focus group discussions and were required to sign an informed consent form before attending the meetings.

### Pseudonymization

Participants’ opinions and perspectives were audio-recorded and transcribed verbatim, pseudonymized and numbered as follows: codes A1 and A2 were assigned to pharmacists, codes I1 and I2 to pharmaceutical company employees, codes E1 and E2 to ethicists, code D1 to a physician of the College for Orphan Drugs, code K1 to a representative of a patient advocacy group for cancer, code C1 to a member of the Belgian Drug Reimbursement Committee, code C2 to a health insurer and code C3 to a member of the healthcare policy cell of the Belgian Minister of Health and Social Affairs.

### Data analysis

Data were analyzed according to the Qualitative Analysis Guide of Leuven (QUAGOL) which is a process consisting of two parts, each subdivided into five steps [41]. In the first part, an iterative process was organized to familiarize with the acquired information and a code tree was constructed. In the second part, data was transferred into the QSR NVivo Release 1.3 qualitative software program and coded by linking quote to concept. All steps were discussed and validated amongst students and researchers before results were finally summarized in a framework matrix. The privacy and confidentiality were guaranteed throughout the entire process and data were stored in accordance to the five-year storage policy of KU Leuven.

## Results

### Exploring alternative financing models for orphan drugs

Ethicists unanimously agreed that financing orphan drugs requires a unique approach that considers inherent issues related to rare diseases which impede patients from receiving treatment. Having specific criteria for orphan drugs would prioritize equal access for all patients by counteracting these disadvantages. Therefore, irrespective of the financing model, some sort of exceptionalism should be incorporated into the design (see Table 1, quote 1).

**Table 1:** Quotes of participants' perspectives on the adoption of an alternative financing model in Belgium

Overarching statement for any financing model	*E1*	*1) “If you start lumping orphan drugs together for reimbursement like the majority of drugs, you will not get anywhere. If you adopt one criterium, a one-size-fits-all, people with special health needs that often experience difficulties, for which they do not have any liability, will be excluded. Not due to their disease but by way of distribution. So, if you want to categorize that disease, you will have to approach it in a different way. Otherwise, they do not stand a chance.”*
Insulated orphan drug fund	*E1*	*2) “An insulated fund would allow separate rules, considering rarity and the challenges of orphan drugs. Is it the only way to do so? I am not yet convinced myself. But it is a way to deviate from the standard that is applied elsewhere and that is certainly not fitting for assessment of orphan drugs.”* *3) “If you consider a privately financed fund, you make patients dependent not on the government, something well-structured, but on the goodwill of the industry. You would wonder whether that is appropriate for the most vulnerable to be at the mercy of a private fund rather than public policy.”*
	*D1*	*4) “How would you compare the reimbursement of a third-line oncologic that prolongs one patient’s life with four months compared to another therapy with five candidates that would obtain a different life quality? Through the same fund? You would still have discussion, isn’t it?”* *5) “Does everyone get access to the fund or do people with five million euros in their bank account get the same treatment as someone who is unemployed?”* *6) “The core is that it is such a mixed population with variations of pathologies. Secondly, for rare diseases and their therapies, there are often many uncertainties. Those are two main elements that you should cover. That would be challenging with something that is fixed for ten years. You will always need something dynamic.”* 7) *“If an orphan drug is prescribed in two or three centers, the files are always complete. It becomes a problem when a particular orphan drug is prescribed in 40 to 50 centers, because then there are a lot of people who have little experience prescribing some of those drugs which makes the files worse. A mechanism should be thought of to limit orphan drug treatments to one or two centers.”*
	*I2*	*8) “I don’t think pharmaceutical companies would favor sponsoring such a fund. Instead, we should plead for an expansion of the medication budget to help as many patients through standard procedures.”*
	*E1*	*9) “If you adopt a system of fair pricing, it would simplify making more budget available. If a disease is life-threatening and of high medical need, those fair prices could be set higher.”*
Emergency fund: The Special Solidarity Fund (SSF)	*C1*	*10) “The SSF should remain an exception. It is case-by-case, so really not for everyone with a rare disease. It is to answer needs when there is no structural solution or to give access to medicines not yet registered. For adults with a rare disease, costs are never fully covered. It is also a limited budget that could run out.”*
	*I2*	*11) “It is a very laborious administrative procedure. Patients need to apply, usually with help of the hospital’s social services, but it is usually not applied for due to the tediousness and the significant delays.”*
Private insurance	*C1*	*12) “Access will not be uniform. Since, in Belgium, we highly consider the principle of solidarity, I do not think it is applicable in our country.”*
	*E1*	*13) “This will *de facto* lead to a two-track policy. There would be decent public healthcare but the moment it becomes expensive and inaccessible, it would be handed over to private insurance. Making it accessible to only those who can afford it or who are administratively resilient enough to arrange it.”* *14) “If everyone participated, that amount would remain relatively low, but if, for example, only 10,000 people register for it, that paints another picture”*

*C1* member of the Belgian Drug Reimbursement Committee; *D1* physician of the College for Orphan Drugs; *E1* ethicist; *I2* employee of pharmaceutical company; *SSF* Special Solidarity Fund

The following sections describe the various orphan drug financing models and their operational preconditions (see Additional file 2, Table 1) that were discussed by focus group participants.

#### Insulated orphan drug fund

Based on Rawls’s Maximin principle, that defines maximizing the minimal situation as morally responsible, ethicists conveyed their interest in an insulated orphan drug fund [42]. Isolation of a predetermined budget in such fund would establish a distinct procedure that accounts for rarity, vulnerability and complexities associated with orphan drugs. According to a health insurer and an ethicist, monetary contributions as a proportion of drug prices from pharmaceutical companies could be legally enforced. Although, such fund would likely be most valuable in countries with restricted public resources (see Table 1, quote 2).

Conversely, most participants expressed that a fund would not necessarily equate to a solution. Excluding a vulnerable group from the standard procedure could also be considered ethically unsound. Discussion on contribution percentages would arise and, in case of private financing, patients would be relying on the industry’s goodwill. Moreover, one physician expressed that in a previous civil society forum, civilians preferred not to separate orphan drugs from other medicines. Also, budgetary constraints and associated challenges such as, eligibility, budget size, allocation, disease heterogeneity and depletion would remain. As the Belgian healthcare system is founded on solidarity, an insulated fund would contradict this principle (see Table 1, quotes 3–5).

Furthermore, a fund implemented as a structural solution for financing orphan drugs, should ideally guarantee continuity in reimbursement in the form of a dynamic, inexhaustible source. An alternative would be to isolate a percentage from the overall drug budget of which the share would be determined as a function of disease rarity or health gains. However, participants preferred to maintain solidarity, through specific attention towards orphan drugs, within the standard reimbursement procedure and reform only that which requires it (cfr. infra). For instance, concentrating orphan drug prescription and treatment in a handful of expertise centers could enable efficient data generation on clinical uncertainties, alleviate workload of the Orphan Drug College and ameliorate quality care for patients (see Table 1, quotes 6–7).

However, some company representatives voiced that the current drug budget should be increased by generally re-evaluating current reimbursement of drugs. In contrast, ethicists expressed that a fair price system, in which a high, but reasonable price of an orphan drug is justified, could free up more financial capital (see Table 1, quotes 8–9).

#### Emergency fund: The Special Solidarity Fund (SSF)

Stakeholders expressed that emergency funds such as the SSF could only efficiently operate for bridging an exceptionally critical period and not as a standard reimbursement procedure for orphan drugs in Belgium. Since access to the fund is tied to strict criteria and limitations, this type of funding is highly unreliable for patient and physician. Furthermore, adults are still faced with significant out-of-pocket costs since not all expenses are covered. It was also felt that the current operation of the SSF is characterized by tedious administration and complex registration. Even though, in 2010, the Belgian Health Care Knowledge Centre drafted recommendations to optimize the SSF, little is known about its realization (see Table 1, quotes 10–11).

#### Private insurance

The majority of stakeholders opposed the adoption of private insurance to finance orphan drugs. According to them, this system is profit-driven instead of needs-focused. Insurers would be prone to demand stellar prices for admission since accommodating orphan drugs is often linked to high costs. Furthermore, it would be highly inefficient for each insurance company to perform their own health technology assessment to determine which orphan drug to take on financially. Ethical debates could arise from admission requirements, such as genetic testing, which would reduce access to these insurances. The popularity of such a scheme for orphan drugs could also be questioned as incidence rates for rare diseases are generally low. Overall, stakeholders were of the opinion that private insurance, similar to an insulated fund, would be incompatible with the foundational belief of solidarity in Belgian healthcare (see Table 1, quotes 12–13).

In contrast, ethicists conveyed that private insurance as an additional system could establish a competitive market that might provide accelerated access to orphan drugs. Solidarity could be upheld if genetic testing, as a prerequisite for coverage, would be prohibited by law. Current complementary insurance in Belgium demonstrates that it is possible to keep costs relatively low. Furthermore, costs could be contained if a large population takes on private insurance. In the end, people would rather have part of their orphan drug expenses funded, with or without a shorter delay, than assume full out-of-pocket costs (see Table 1, quotes 14).

### Access schemes for fast-track delivery of orphan drugs

Focus group participants discussed a number of access schemes for orphan drugs and their operational preconditions (see Additional file 2, Table 2) as described in the following sections.

**Table 2:** Quo﻿tes of participants' perspectives on access schemes for fast-track delivery of orphan drugs

Early Temporary Authorization (ETA): compassionate use program/medical need program (CUP/MNP)	*I1*	*15) “We checked how many percent of the compassionate use programs were approved orphan drugs, and that was one third of the compassionate use programs approved by the agency of orphan diseases.”*
	*D1*	*16) “As of yet, there has also been a problem with the data collection associated with it.…I hear all sorts of things, I hear there are confidential issues involved. I also hear that the prescribers do not want to go to the trouble of collecting that data. Companies certainly do not want to go through all of that administration for data collection. There is a lot of ambiguity at the moment.”*
	*C1*	*17) “It is necessary to decide who is responsible to collect data. So far, it is one that says, it is the companies, the other one says, it is the doctors and a third one says, it is the authorities but, in the end, insufficient data is collected. Until it is clear, maybe legally or another way, who is responsible for it, I am afraid nothing will happen…And also define who is the owner of the data…that owner also must be willing to share the data.”* *18) “In day-to-day practice, I don't think everyone uses the same measurements necessarily, so it's very difficult to compare results between patients and draw a conclusion about that kind of data. It is also a kind of real-world data, hey, there must be some form of standardization to be able to interpret this data.”*
	*D1*	*19) “Well, it's actually more complicated, I mean we tried to make it easier, the intention is that a parallel submission will be made to the FAMHP and the RIZIV/INAMI. At the same level, in collaboration with the FAMHP, the ethics committee would also intervene. However, that is something new, why do I mention it, because we also proposed it at European level and there was quite a bit of interest shown for it.”*
	*I1*	*20) “Yes, it would be important to have an electronic system. Also, for the orphan drugs with a College because we support the collection of wearables.”*
	*A2*	*21) “If that data then plays a major role, then we also have to be sure that it is correct, right? Yes, those systems must be validated, otherwise they are really not valuable.”*
	*D1*	*22) “But if you link that to conditional reimbursement, then maybe it could be implemented, huh. Maybe not such a bad idea, that you ask the patient to fill in data 2–3 times a year for 5 or 10 min and that that is one of the conditions that he/she gets his/her medication. I don’t think these should be absolute conditions, but we should ask nicely anyway…a large framework has to be created to realize that, and if that is the case, I can imagine that the patient is invited to register data anonymously, of course. There needs to be a legal framework. Nowadays, it seems that there are mainly discussions since GDPR is unclear.”*
	*E1*	*23) “I think with permission you never have problems, if the patient gives the informed consent. Suppose that something makes a real difference scientifically and as a society you invest disproportionate resources into it, then from an ethical point of view I think that is also a discussion that you should dare to have in order to say that we expect something in return. I also think that if you use solidarity in the context of very expensive resources… if it is really essential to demonstrate whether something provides sufficient efficacy and return to pay for it as a society, then I think you can discuss it every now and then.”*
	*K1*	*24) “I think the philosophy is also not to centralize it all, but to ensure that the databases that are available can communicate with each other.”*
	*C1*	*25) “But I think one of those solutions that are shared and discussed nowadays, is that we would actually register real-world data so that a real analysis could be done after a certain duration of treatment so that a better decision could be made in the end…The Orphan Drug Colleges would be a good opportunity for that …Well, in fact, it has to be decided upfront what data should be collected, for how long, by who…so that we have quality data in the end that can be interpreted.”*
Early Temporary Authorization (ETA): Early Temporary Reimbursement (ETR)	*I1*	*26) “…the idea of choosing the amount was to cover the logistics costs…and that amount is relatively low, but is to compensate for the administrative burden of the industry …We would like to improve the ETR and make it more appealing. But now it's relatively burdensome and doesn't bring much more than a CUP or MNP, so if the companies have to choose now, it's easy in fact, CUP or MNP…”*
	*D1*	*27) “We have been very dynamic, in the beginning it was a compensation, a lump sum, which was initially seen as a kind of foreshadowing of the final reimbursement by the industry, it was then made clear that it was not the case and then it also disappeared…”*
	*I2*	*28) “Yes, I can only speak for the industry, we are of course in favor of quick and temporary reimbursement until a final decision has been made about reimbursement. So, the principle (of ATU) is something that we as an industry are advocating for. Of course, the scope has to be aligned. Which medicines are now eligible for this? It goes without saying that you cannot reimburse all medicines the same way.”*
	*D1*	*29) “in ideal circumstances that (unmet medical need) list would not only be fed by the industry, but also by the unmet need defined elsewhere, but yes, as long as the minister does not define what unmet need is…”*
	*C1*	*30) “No, sorry to say, but I think yes, there should be suggestions but from the field, that is, from doctors, but also from the health insurance fund because they see what the medical need is in society.”*
Off-label use	*I2*	*31) “It is an increasing phenomenon…drugs that work *via* a certain pathway and if that pathway applies to a tumor in the lung or in the colon…doctors believe that if the pathway is the same for a tumor in a different location, I would like to try that (same medicine) with this patient, but there is no registration for it.”*
	*A2*	*32) “Sometimes off-label use is already in the guidelines…then it is clear that everyone agrees with it.”* *33) “I think the NIHDI plays a big role in off-label use. Sometimes it is not reimbursed and sometimes it is. If it is not reimbursed, it is often financially difficult to establish off-label use.”* *34) “It will always be difficult to report the negative effects, but we actually need that.”*
	*D1*	*35) “But not the off-label use and that is the problem…evaluations of all systems have to be made and again about GDPR…who should collect which data, who is allowed to…who is entitled to that data and who owns it and so on.”* *36) “…what we are reflecting about are those centers of expertise, reference centers, after the European example…for those kinds of very rare diseases off-label use could be permitted in only one or two centers in Belgium. This could be done with other people, so that there is peer control. There must be, so to say, a joint database. Or at least have some sort of yearly reporting in an expertise center.”*
	*I2*	*37) “I think that there should be partial responsibility between authorities and companies…And, of course, you could also think about a shared responsibility for financing. Now, the doctor is completely dependent on the goodwill of the company and we are aware that some companies are more willing to participate than others.”* *38) “…evidence should also be collected, which could perhaps be part of the answer…So that, in case of off-label use, data is also produced by those doctors and that progress can be made, that evidence is created.”*
	*C2*	*39) “This may be possible through the FAMHP. A legal framework can be drafted by the FAMHP in which, based on an evaluation, is determined which (off-label use) we now think is justified.”*
	*I2*	*40) “But that's why it might not be a bad idea what C2 proposes, that is possibly one option. That the limited evidence that there may be of off-label use, because I think we agree that it is limited compared to when there is a market authorization, that you let an independent body judge it. When you talk about the FAMHP, I have my reservations since the FAMHP currently works on a fee for service basis…a fee of 11 000 euros should be paid for an evaluation, which is not free, I would say. Another option could be…similar to the Netherlands, that scientific associations would do that evaluation. For example, that doctors who prescribe off-label are evaluated for impertinence by scientific association…so basically, the peers. Then one would have to deal less with that financial element and perhaps be even closer to practice and the people who are really aware of the latest good practices, than when you would involve FAMHP.”* *41) “What is the representativeness if you have two patients in Belgium? It would be better to have an international registry.”*
	*C2*	*42) “But then again, we have to be careful to do it in controlled circumstances, because that's the problem right now. Certain companies acquire those (smaller businesses), then re-exploit the monopoly position making it to become extremely expensive, whereas before, it could be used unnoticeably at a reasonable price.”*

*A2* pharmacist; *C1* member of the Belgian Drug Reimbursement Committee; *C2* health insurer; *D1* physician of the College for Orphan Drugs; *E1* ethicist; *K1* representative of cancer patient’s advocacy group; *I1/I2* employees of pharmaceutical company; *ATU* autorisation temporaire d’utilisation; *CUP/MNP* compassionate use program/medical need program; *ETA* Early Treatment Authorization; *ETR* Early Treatment Reimbursement; *FAMHP* Federal Agency for Medicines and Health Products; *GDPR* General Data Protection Regulation; *KCE* Belgian Health Care Knowledge Centre; *NIHDI* National Institute for Health and Disability Insurance

#### Early Temporary Authorization (ETA)

##### Compassionate use program/medical need program (CUP/MNP)

In the context of orphan drugs, stakeholders were of the opinion that CUP and MNP are positive initiatives to provide patients with essential treatment at an early phase while generating clinical evidence. Besides, one third of drugs delivered through ETA receive orphan designation from the European Medicines Agency. Moreover, contrary to off-label use, more substantial preliminary evidence on the drugs exists. ETA is, namely, only approved under condition of ongoing clinical trials or market authorization application at the European Medicines Agency and after a benefit-risk analysis by the Federal Agency for Medicines and Health Products (FAMHP) (see Table 2, quote 15).

However, stakeholders argued that these programs in Belgium need improvement as some hurdles hinder their adoption. For instance, prior permission from the FAMHP and an opinion from the ethics committee and the Commission for Human Medicines is required in order to proceed to an application for CUP or MNP at NIHDI. Involving multiple government bodies in such a process is often considered expensive, tedious and time-consuming. Recent amendments to the CUP/MNP procedures following an update of the Royal Decree on medicines for human and veterinary use, have additionally complicated the process. Furthermore, physicians were often hesitant to decide on a specific company or clinical trial for ETA, as a variety of options often exist and the company should consent to initiating such program. Moreover, General Data Protection Regulation (GDPR) for ETA, in terms of confidentiality and data capture, is unclear. In turn, the collection of clinical evidence is currently uncoordinated and privacy issues remain unsolved. Redundant or inaccurate information is then assembled into multiple systems which makes data extraction complicated and burdensome (see Table 2, quotes 16–18).

To reduce administrative burden and accelerate the process, parallel submission at FAMHP, NIHDI, the ethics committee and the Commission for Human Medicines of the application dossier for CUP/MNP should be facilitated. Stakeholders suggest to legally extend responsibility of selecting an adequate program to company and authorities. This could stimulate CUP or MNP initiation by alleviating decision fatigue of physicians and leveling the playing field. Another remark was to possibly implement these programs at European level instead of national level. Increased efforts towards data capturing of clinical outcomes should be aimed for by fostering a legal framework with clear instructions. Raising awareness amongst people living with a rare disease on the value of information sharing for high-cost orphan drugs, could also increase informed consent rates for data capture during early access programs and generate additional clinical evidence. Prior to initiation of ETA, an agreement on which parameters to analyze should be reached to ensure highest quality of real-world evidence. However, a first step to defining these parameters would be to guarantee a well-structured list of current reimbursed drugs requiring prior authorization by a physician of the health insurance fund (this is known as Chapter IV reimbursed drugs in Belgium). Current available information in databases should be gathered, pseudonymized, fine-tuned and electronically connected by experts. To ease data collection, automation and digitalization should be prioritized while disease treatment and drug administration should be limited to selected expertise centers. Electronic devices such as wearables could further aid with collecting data, however both devices and data should be analyzed and validated by specialists, such as those from the Colleges of Orphan Drugs. People living with a rare disease could be closely involved during the application process and neglect could be avoided by creating patient-friendly, electronic dossiers (see Table 2, quotes 19–25).

##### Early Temporary Reimbursement (ETR)

Pharmaceutical companies rarely apply for ETR when offering an orphan drug through CUP or MNP. Stakeholders were convinced that implementation of ETR is hampered by the complicated eligibility criteria for a positive reimbursement decision, the layered application process but also the lack of familiarization with the concept. Moreover, pharmaceutical company employees emphasized that the proposed compensation is a fixed and modest amount to cover logistic costs, which is negligible compared to the costs of offering the orphan drug (see Table 2, quotes 26–27).

In this light, stakeholders supported the prospects by the Belgian Parliament to reform and simplify the ETR procedure. Some suggested to adjust the current ETR to the French ATU system that has well-established data capturing and provides the companies with a customized reimbursement amount, others did not support this idea as Belgian regulations do not allow any correlation of the temporary compensation with the final reimbursement amount paid by NIHDI. As an alternative, the physician proposed to reimburse orphan drugs that are accessed through ETA subject to the condition of data collection. One stakeholder voiced that research in other disease areas could be stimulated to add suggestions from physicians, patient organizations and health insurance, beyond the industries requests, to the unmet medical needs list. In case a developed drug addresses unmet medical need, an agreement with the company could more easily follow (see Table 2, quotes 28–30).

#### Off-label use

Many orphan designations originated from off-label prescriptions, therefore, stakeholders were convinced that off-label use could be valuable to affordably accommodate unmet medical needs. However, improved regulations and stricter monitoring should be adopted. Reimbursement could also be requested without registration at EMA, if some evidence on small cohorts gathered by scientific institutions, such as the Belgian Health Care Knowledge Centre (KCE), is available (see Table 2, quotes 31–32).

Moreover, it is clear that reimbursement of off-label use is not guaranteed. In turn, financial hurdles arise when requests are denied and patients are subject to full out-of-pocket costs. In those instances, real-world data is improperly followed-up and not digitally recorded into a register. This comes with a risk of infrequently reporting adverse events, not detecting abuse of practice, difficulties in evaluating efficiency of the orphan drug, non-specialist’ prescriptions and discrepancies in methods of data gathering as no consensus on GDPR currently exists. Although, the company is responsible for the conformity and quality of the product, physicians are fully liable for its prescription and are therefore hesitant to offer off-label use. Furthermore, a pharmacist emphasized the lucrative practice of drug-repurposing by pharmaceutical enterprises where drugs, that were relatively cheap and proven efficient in off-label use, turn extremely expensive after orphan designation (see Table 2, quotes 33–35).

Similar to ETA, stakeholders expressed that management of off-label use should occur by appointing specific expert facilities for its adoption in therapy. This would enable peer review, continuous evaluation, communication of clinical evidence and advice on usage. Alternatively, a legal framework could be set up that would allow the FAMHP, an independent scientific body or a doctor’s association to formulate guidelines or advice on use. The latter could alleviate the medical liability on physicians and yield valuable qualitative data. Off-label use could be further encouraged if financial responsibility is distributed amongst the company and health authorities. An effort towards data collection should be made nationally through improved structuring of medical dossiers and referencing KCE trials for clinical evidence, although these may be of limited power. Qualitative real-world data should be standardized, linked and centralized into a transnational register that is closely monitored. Finally, stakeholders advocated for stimulating drug-repurposing in a controlled environment to prevent soaring prices (see Table 2, quotes 36–42).

## Discussion

This qualitative study compiles multidisciplinary expert opinions on trade-offs between different access schemes (i.e. early access schemes and off-label use) and various financing models (i.e. insulated fund, private insurance, or emergency fund) for orphan drugs in Belgium. Stakeholders substantiated why alternative financing models would or would not be applicable in Belgium and also highlighted which and how processes for early access should be preserved or ameliorated.

Although ethicists found that an insulated fund would be able to highlight orphan drugs considering their neglected position amongst other drugs, it was generally determined that solidarity, the main principle of Belgian healthcare system, would be disregarded in such fund. Complete isolation of orphan drugs on a smaller scale would reproduce the same budgetary concerns as the conventional reimbursement system in terms of budget size, depletion, allocation, disease heterogeneity and eligibility. These issues have previously been encountered in countries like England and Scotland, that had to initiate several reforms of their insulated funds [18, 21]. Another issue was whether the insulated fund needed to be funded by government and thus the general public, industry or both. Representatives of pharmaceutical companies conveyed that the industry would not be in favor of sponsoring such fund as discussion on contribution percentages would arise. This could be argued as in countries like Italy and Scotland, this system has been well-established by law [16, 19].

Private insurance was generally perceived as profit-driven based on examples of its functioning in North-America and thus inadequate for a system based on solidarity [43]. Experts were skeptical about its membership ethos requiring sensitive or personal information-sharing but also about elevated fees dependent on the number of affiliated members. An emergency fund, such as the SSF, was considered incapable to be reformed into an insulated fund that would be fully responsible for the financing of orphan drugs. This emergency funding is unreliable (as it is often temporary), hindered by many restrictions such as a fluctuating, limited budget, and can thus only operate on a small scale.

Overall, stakeholders believe that it would be better to improve the functioning of current established systems (in terms of efficient data generation, decreased administrative burden, more financial capital for orphan drug expenditure) instead of introducing new ways for financing orphan drugs. It could be argued that this might not be the ideal approach towards orphan drugs as this thought process may be influenced by human tendencies to stick to the status quo [44, 45]. It could, furthermore, also be that the proposed models were incompatible with compulsory social insurance in Belgium [46, 47]. The insulated fund was, namely, based on examples in Italy and the UK, which have a general taxation system, and on the USA and Canada which have a strong private insurance system [23, 24, 46]. However, optimization of current processes is not always evident as shown by the KCE initiative to improve the SSF more than ten years ago, with little to no implementation today [36].

This research additionally reveals insights on the implementation of early access schemes and their conditions, such as CUP/MNP with ETR and off-label use, for fast-track delivery of orphan drugs to people living with a rare disease. Stakeholders unanimously consented that early access to orphan drugs is a benefit to the treatment of rare disorders that often need immediate attention. It was obvious that these schemes must be kept, however major reforms should be introduced. For CUP/MNP and ETR, the administrative burden should be reduced to make the programs more attractive. For off-label use, a legal framework should be defined to ensure its proper use in adequate instances. An overarching precondition that frequently reoccurred in the discussions, was the importance of quality, robust and digital data collection. This finding corroborates recent survey results of encouraging stakeholders to consult registries in decision-making [48]. Prior to initiating therapy with an orphan drug, a protocol with clear questions and instructions on what evidence to collect should be drafted. Selecting a handful of expertise centers permitted to treat rare diseases with orphan drugs or experiment with off-label use, could foster a more coordinated environment for monitoring and data gathering.

Another important aspect that was mentioned, was the need for frequent and efficient communication through standardization of data measurement but also strategically linking current existing databases. The European Commission defined some common data elements for rare disease registries, however, practical actions are yet to be taken [49]. The Danish Health Data Authority, which successfully established comprehensive and quality assured health data in Denmark, might serve as an example for future measures in setting up a centralized health data authority for rare diseases [50, 51]. Upscaling these initiatives to European level would most probably increase the representativeness and meaningfulness of collected data for rare diseases often affecting just a small population nationally. These benefits of cross-country collaboration on healthcare have been shown by European establishments such as the International Horizon Scanning Initiative and European Reference Networks [52, 53].

Although this qualitative study evaluated financing models and early access schemes for orphan drugs from the perspective of the Belgian healthcare system, it has also produced some relevant take-home messages for an international audience. In the case of cross-country collaboration and healthcare, it will be important for all participating EU countries to have strong and coordinated data capture, regardless of the established financing models and access methods for orphan drugs, for seamless communication between healthcare systems. This may be especially relevant in the context of the often, small population suffering from a particular rare disease. Stakeholders outlined several suggestions to improve data collection. For countries such as Austria, France, Germany and Luxembourg with healthcare systems based on compulsory social insurance, similar to Belgium, an insulated funds or private insurance might not be the first choice [46]. However, the underlying element of a separate approach in an insulated fund and the generation of more competition (and thus potentially improved access to orphan drugs) in the case of private insurances, are positive aspects that should be maintained or prioritized also in these systems with solidarity at the basis. In contrast, countries which finance healthcare through general taxation, such as England, Scotland, Italy or have a system of private insurance in places like Canada and the USA, can draw from the criticism stakeholders have expressed during the discussions [23, 24, 46]. Are current methods to counteract depletion or scarcities in resources within funds such as New Medicines Fund, Cancer Drug Fund and AIFA robust and sustainable on the long run? Are there ways to control price setting and prevent unaffordable prices of life-saving orphan drugs in a climate where private insurances predominantly finance healthcare?

For this study we were able to gather various experts on orphan drugs from different fields for three extensive focus group discussions, which provided relevant information on accessibility and funding of orphan drugs. However, the conduct of our study was also subject to some limitations as focus group discussions were held online due to the ongoing global health crisis. This meant that communication might have been affected by technical issues relating to quality of internet connection and recording devices. Moderating a discussion in such environment was also challenging. Some participants were generally more outspoken than others, which meant that not all perspectives might have been equally conveyed. Moreover, the output from the focus groups was partly determined by the posed questions but was naturally also limited by the knowledge of the involved experts. The pandemic might also have influenced responses as, for certain stakeholders, other priorities may have been at play. Finally, it is worth mentioning that the involved physician had already been retired at time of conduction of the research.

Future research should involve experts on orphan drugs from all over European countries to delve into formulating concrete and practical actions for a legal framework on cross-country collaboration at EU level. This orphan drug framework should address, inter alia, coordinated and digitalized data capture, GDPR, terms and conditions for off-label use and the possibility of a centralized early access scheme.

## Conclusion

This study revealed that alternative financing models such as an insulated fund and private insurance for orphan drugs were perceived by stakeholders to conflict with the foundational principle of solidarity in the Belgian healthcare setting. It, furthermore, showed lack of adequate data collection, administrative burden and poor communication to be the main hurdles to early access, through compassionate use, medical need program or off-label use of orphan drugs. We believe that the concepts and gaps identified in this study, may stimulate other jurisdictions to review and improve their models of early access and financing to foster a more sustainable access to orphan drugs.

## Supplementary Information

**Additional file 1**. Focus group discussions guide.

**Additional file 2**. Participants’ preconditions.

## Data Availability

Data are available upon request to the corresponding author.
