# Supplementary material for: Exploring alternative financing models and early access schemes for orphan drugs: a Belgian case study
Source: Orphanet J Rare Dis. 2022 Dec 9;17:429. doi: 10.1186/s13023-022-02571-8 (PMC9733299; doi:10.1186/s13023-022-02571-8)
Supplement: Supplementary file 1 — Additional file 1. Focus group discussions guide. [file 13023_2022_2571_MOESM1_ESM.docx]

**Appendix 1: Focus group discussion guides**

Focus group discussion 1:

- Could an alternative payment model or access method for orphan drugs be adopted in Belgium?
- Examples in other countries: compassionate use in France and Slovakia and off-label use (early access schemes); an insulated fund such as AIFA 5% fund in Italy or a private insurance or health plans in Canada and the United States of America (payment models)
- What are the advantages and/or limitations of private insurance or health plans? Could this model be adopted in Belgium?
- What are the (dis-)advantages of an insulated fund? Could this model be implemented in Belgium?
- What are the (dis-)advantages of off-label use?
- What are the (dis-)advantages of compassionate use/unmet medical need program?

Focus group discussion 2:

*Insulated fonds*

- From what perspective should the fund be created? Rather patient-oriented (e.g. the Belgian SSF, a yearly fixed and limited amount) or rather reimbursement-oriented (e.g. UK’s cancer drug fund, based on orphan drugs percentage for which an amount adjusted for rarity and health gain is isolated)?
- What is your opinion on the special solidarity fund and its framework? Should it be kept as is or should some things be adapted/ameliorated?
- If orphan drugs remain evaluated through the conventional Belgian reimbursement system (CTG), should there be adjustments in terms of criteria? Should corrections for rarity or health gain be considered? To what extent is this currently happening?

*Off label use*

- How would you improve regulation for off-label use? Would a GDPR-friendly framework for notifying and reporting off-label use be beneficial?
- How could data collection of off-label use be done efficiently? Could the cancer registry serve as an example for setting up a registry for off-label use of orphan drugs?
- What do you think of physician guidelines on off-label use?
- (Previously we discussed that tracing of off-label use is not evident since no reimbursement is available.) Would a refund or compensation ameliorate the adoption of off-label use? For example, by conditionally refunding off-label use only if the company properly registers and validates all data that is then verified and assessed by for instance a College of Experts.

*ETA/ETR*

- Under (ETA and) ETR, a fixed temporary compensation can be requested by the companies. However, ETR is not very popular. Could a compensation based on the projected price of the medicine at market launch, as done in the French ATU system, also be adopted in Belgium?
- How would you optimise data collection for ETA?

*International*

- What is your opinion on creating an Unmet Medical Need list with priority diseases at European level (following the example of the list in Belgium) for which the EU would reimburse therapies and invest in development? (If the EU then transparently communicates their budget, government agencies could have a predetermined price to refer to and companies would have an extra incentive to develop new therapies.)
- Would it be possible to connect the network of expertise centres internationally and map out to which reimbursement criteria an orphan drug is subject in a specific country? This way, perhaps reimbursement in every Member State would not be necessary since cross-border health care would give access to treatments. Would this be feasible?

Focus group discussion 3:

- Are the current exceptional considerations (exempted from economic evaluation, reimbursed despite uncertainties) for orphan drugs within the conventional reimbursement procedure in Belgium sufficient? Would it be logical to consider heterogeneity and different rare disease categories in reimbursement?
- How would you increase the pharmaceutical budget to meet the needs of all patients through the conventional system? Would re-evaluation of current reimbursed medicines be a solution?
- The last focus group also dealt with managed entry agreements, these are often extended although initially intended as temporary. What is your take on that? Is it actually useful to extend such agreements and are uncertainties then solved?
- Which criteria are considered by the Orphan Medicines Board to decide on individual reimbursement? Would making this process transparent speed up the reimbursement procedure?

*Insulated funds - SSF*

- Followings questions can be asked about both BSF and internal solidarity fund:

How is the limited budget of the SSF allocated considering the mix of pathologies and uncertainties about therapies? What happens when the budget is exhausted and patients do not receive a compensation through this ‘last resort’ safety net?

- Is there also a reimbursement for disease care beyond the actual medicine?
- Which recommendations from the KCE report on ‘Optimization of the operating processes of the SSF’ have been implemented or optimized and which have not and are still relevant today? (1)

*Early access schemes*

- It was mentioned that ETA/ETR has not been a great success thus far and that plans have been made by the parliament to adjust the schemes. Any updates on what the concrete plans are?
- ETA can only be requested for a specific condition on the Unmet Medical Need list, would expanding this list encourage the demand for ETA and ETR?
- Who compiles the Unmet Medical Need list currently?
- Is data collection under ETA mandatory or could it be made a condition for ETA? Could a patient be required to fill in a questionnaire in order to receive a treatment, for instance?
- Why do pharmaceutical companies rarely request a compensation for ETA through ETR? Could you identify any gaps or issues?

*Off-label use*

- Should off-label use liability and follow-up be spread across authorities, company and prescribing physician? If so, how would this be done efficiently?
- How to centralize available data that is dispersed over different platforms (sickness funds, companies, physicians) and even internationally in one central portal or register? More concretely, how to define which conditions must be met and what information is registered considering GDPR register?
- How can we ensure the prescription of a particular orphan drug solely in a limited number of expertise centres so that submitted files to the College are complete for evaluation and access to orphan drug is accelerated? Or perhaps better, how can we ensure that centres of expertise collaborate to deliver best treatment and care?
- How to raise more awareness of the actual cost of orphan drugs amongst prescribing physicians?
- What does providing incentives in controlled circumstances (so as to avoid extreme price monopoly in case of repurposing) for further development of ‘hopeful’ results from off-label use look like?

References

1. Guillaume P, Moldenaers I, Bulté S, Debruyne H, Devriese S, Kohn L, et al. Optimalisatie van de werkingsprocessen van het Bijzonder Solidariteitsfonds. Health Services Research (HSR). Brussel: Federaal Kenniscentrum voor de Gezondheidszorg (KCE); 2010. Report No.: 133A.
