# Supplementary material for: Exploring alternative financing models and early access schemes for orphan drugs: a Belgian case study
Source: Orphanet J Rare Dis. 2022 Dec 9;17:429. doi: 10.1186/s13023-022-02571-8 (PMC9733299; doi:10.1186/s13023-022-02571-8)
Supplement: Supplementary file 2 — Additional file 2. Participants’ preconditions. [file 13023_2022_2571_MOESM2_ESM.docx]

**Appendix 2: Participants’ preconditions**

**Table 1:** Participants’ preconditions for adoption of a financing model in Belgium

| ***This Financing Model*** | could be adopted if it considers the following: |
| --- | --- |
| Any model | - Exceptionalism in the form of specific criteria |
| Insulated OD fund | - Monetary contributions (as a proportion of drug prices) from pharmaceutical companies or isolation of a percentage (of which the share is determined as a function of disease rarity or health gains) from the overall drug budget - Continuity in reimbursement in the form of a dynamic, inexhaustible source - Orphan drug prescription and treatment in a handful of expert centers to enable efficient data generation |
| Emergency fund (SSF) | - In exceptionally critical cases per individual and not as general, standard reimbursement |
| Private insurance | - Genetic testing as a prerequisite for membership prohibited by law to uphold solidarity - General adoption to contain membership costs |

*OD*, orphan drug; *SSF*, Special Solidarity Fund

**Table 2:** Participants’ preconditions for adoption of an early access scheme in Belgium

| ***This Early Access Scheme*** | could be adopted if it considers the following: |
| --- | --- |
| ETA |  |
| → CUP/MNP | - Parallel submission at FAMHP, NIHDI and the ethical committee - Legal responsibility not only of physicians but extended to company and authorities - Implemented at European level instead of national level - Data capturing of clinical outcomes through a clear, legal framework - Raising awareness of data collection amongst patients - High quality real-world evidence by agreeing on a well-structured list of reimbursed drugs requiring prior authorization and on which parameters to analyze - Electronic databases in which information is gathered, pseudonymized, fine-tuned and electronically interconnected - Automation (via wearables), digitalization and validation - Patient-friendly, electronic dossiers allowing close involvement of patients |
| → ETR | - Reformed and simplified procedure that allows customized reimbursement - Reimbursement only if data collection allowed - Unmet medical needs list includes, next to pharmaceutical company requests, suggestions from physicians, patient organizations and health insurance |
| Off-label use | - Improved regulations and stricter monitoring - Specific expert facilities for peer review, continuous evaluation, communication of clinical evidence and advice on usage - Independent scientific body or doctor’s association to formulate guidelines or advice on use and thus alleviate medical liability of physicians - Financial responsibility distributed amongst company and health authorities - Valuable qualitative data collection in structured medical dossiers - Standardized data linked and centralized into a transnational register - KCE trials for clinical evidence - Controlled drug-repurposing to prevent soaring prices |

*CUP/MNP*, compassionate use program/medical need program; *ETA*, Early Treatment Authorization; *ETR*, Early Treatment Reimbursement; *FAMHP*, Federal Agency for Medicines and Health Products; *KCE*, Belgian Health Care Knowledge Centre; *NIHDI*, National Institute for Health and Disability Insurance
